# Visual short-term memory for crossed and uncrossed binocular disparities

**DOI:** 10.1371/journal.pone.0312202

**Published:** 2024-10-22

**Authors:** Vanda Ágnes Nemes, János Radó, Diána Fülöp, Eszter Mikó-Baráth, Imola Hamvas, Gábor Jandó, Péter Buzás

**Affiliations:** Institute of Physiology, Medical School, University of Pécs, Pécs, Hungary; The Ohio State University, UNITED STATES OF AMERICA

## Abstract

Previous work on visual short-term memory (VSTM) has encompassed various stimulus attributes including spatial frequency, color, and contrast, revealing specific time courses and a dependence on stimulus parameters. This study investigates visual short-term memory for binocular depth, using dynamic random dot stereograms (DRDS) featuring disparity planes in front of or behind the plane of fixation. In a delayed match-to-sample paradigm, we employed four distinct reference disparities (17.5’, 28.8’ either crossed or uncrossed) at two contrast levels (20%, 80%), spanning interstimulus intervals (ISI) of up to 4 s. Test stimuli represented a range of equally spaced values centered around the reference disparity of the ongoing trial. In addition, the impact of a memory masking stimulus was also tested in a separate experiment. Accuracy and point of subjective equality (PSE) served as performance markers. The performance, indicated by the accuracy of responses, was better for smaller reference disparities (±17.5’) compared to larger ones (±28’), but both deteriorated as a function of ISI. The PSE demonstrated a consistent shift with increasing ISIs, irrespective of the magnitude of the initial disparity, converging gradually toward the range of 20–22’ and deviating from the reference disparity. Notably, the influence of masking stimuli on the PSE was more marked when the mask disparity diverged from the reference value. The findings from our study indicate that the retention of absolute disparity in memory is imprecise, it deteriorates with retention time or due to perturbation by dissimilar masking stimuli. As a result, the memory trace is gradually replaced by a default depth value. This value could potentially signify an optimal point within low-level perceptual memory, however, our results are better explained by perceptual averaging whereby the visual system computationally derives a statistical summary of the presented disparities over time. The latter mechanism would aid in the computation of relative disparity in a dynamically changing environment.

## Introduction

Successful engagement with the environment requires the complex ability to perceive, integrate and temporarily store relevant visual information. These processes are critical to interpret and respond to the dynamic challenges presented by our 3D surroundings [1, 2].

Visual short-term memory (VSTM) has been investigated extensively for many visual dimensions, such as color, spatial frequency, contrast, and velocity [3–9]. It has been argued that the neural mechanisms for the short-term storage of such elementary features are similar and closely connected to perceptual mechanisms, hence the name perceptual memory. Perceptual memory transiently stores behaviorally relevant information in a fragile form, exhibiting a decline in precision over time [2]. Studies focusing on the short-term storage (0–30 s) of visual features reveal a time-dependent decay of information for some of the attributes such as color and contrast. However, other parameters, like spatial frequency, exhibit remarkable stability and can only be interfered with by memory masking stimuli in a psychophysical experimental paradigm [2, 6, 7, 9, 10]. These masking studies have revealed that the storage of visual attributes is organized into functionally separate, feature specific channels. Within each of these dimension specific components, there is an interference between similar memory items, and an independence among stimulus attributes that are substantially different [4, 6–8, 10–13]. These findings show many similarities with previous research data about perceptual processing, thereby providing further evidence in favor of the essential role of visual cortical areas in the short-term storage of visual information [2, 13–16].

Stereopsis, a fundamental aspect of visual processing, takes advantage of disparity information from the differences in the binocular visual input caused by the horizontal separation of the eyes. The brain employs this disparity to compute depth and three-dimensional structure, thereby facilitating the perception of the three-dimensional spatial relationships between the self, objects, and their environment [17]. Parallel processing emerges as a fundamental organizational principle for disparity, as demonstrated by adaptation experiments [14, 18–20]. Various studies demonstrate distinct processing mechanisms for near (crossed) and far (uncrossed) disparities, with a bias for near disparities evidenced by lower detection thresholds [21], faster reaction times [22], decision reaction times [23], and lower duration thresholds [24–26]. Moreover, sensitivity to crossed disparity also develops earlier in infancy [27]. Similarly, a dichotomy exists in the detection of smaller (fine) and larger (coarse) disparity information [28–30].

VSTM for binocular disparity is probably a key component in analyzing the spatial arrangement of the environment and guiding behavior. Similarly for other visual attributes, the low-level neural substrates involved in stereoscopic processing, including the primary visual cortex, are plausible candidates contributing to the encoding and maintenance of binocular disparity in VSTM [31].

One potential mechanism affecting the memory trace during prolonged retention is perceptual averaging. It refers to the cognitive process in which the brain combines and integrates multiple sensory inputs or perceptual signals to create a unified or averaged representation. This can occur in various modalities, such as vision, audition, or touch and as a result, a more stable, coherent, and reliable perception of the external world is formed, and it can have a significant role in memory formation. This process also helps the brain overcome noise, variability, or uncertainties in individual sensory inputs. The phenomenon has been demonstrated in various visual psychophysical studies, whereby a representation of the statistical average of the items presented over successive trials is built up [32–35].

As outlined in the study of Huang and Sekuler [36], task-irrelevant stimuli have an impact on the early encoding process. Specifically, they proposed that participants stored a weighted average of both relevant and irrelevant memory items during trials, resulting in the formation of a collective or typical representation, which in turn, influenced the recall of the task relevant item. These findings suggest that the neural mechanism of perceptual averaging overlaps with, or it is identical to the mechanism of VSTM.

When a memory-masking stimulus is introduced into a trial, it disrupts performance, despite its lack of relevance to the current task. The remembered parameter can exhibit a bias towards this irrelevant stimulus, referred to as a "pulling effect". This phenomenon also highlights the impact of perceptual averaging in VSTM situations as it can result in uncertainty regarding the contents of VSTM. Consequently, the memory representation can be further modified, particularly as memory intervals increase [35].

The current study investigated visual perceptual memory for binocular depth, utilizing dynamic random dot stereograms (DRDS). This stimulation technique allows the presentation of image pairs with features coded in binocular disparity, that are perceptible solely binocularly, and with intact stereopsis [37, 38]. We explored how the storage of disparity information changes with absolute disparity, retention time and contrast. In another experiment, we evaluated the modifying effect of additional masking stimuli during storage time.

**Fig 1:**
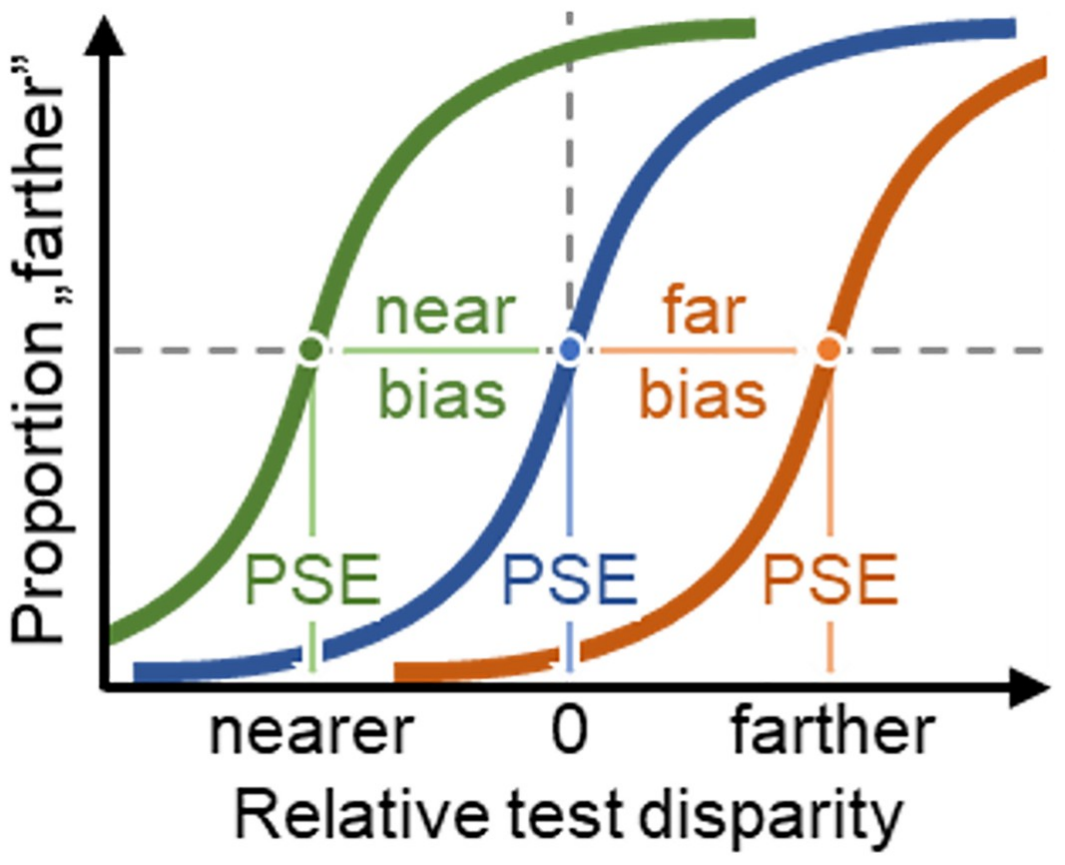
Theoretical outcomes of a delayed discrimination experiment for disparity memory. The test disparity where participants choose nearer and farther with equal probability (horizontal dashed line) in the test phase of the trial is the point of subjective equality (PSE). If disparity is retained accurately in memory, the PSE is expected to correspond to the reference disparity to be memorized (blue). A shift of the memory trace either towards nearer or farther disparities results in corresponding shifts of the PSE (shown in green and orange, respectively). ‘Relative test disparity’ on the abscissa refers to the difference from the reference disparity, which is different from what is usually meant by ‘relative disparity’ in the literature (see Fig 2 and Materials and methods for details).

The main tool of our investigation was a delayed discrimination paradigm whereby participants had to judge if a test stimulus was nearer or farther than the reference depth kept in their short-term memory. If disparity was retained accurately, the point of subjective equality (PSE) between the recalled depth and the test stimulus was expected to correspond to the reference depth (Fig 1). We hypothesized that a shift of the memory trace either towards nearer or farther disparities would result in corresponding shifts of the PSE.

Our findings indicate that disparity memory is imprecise. As retention time increases, the disparity memory trace gradually shifts towards a default value. This effect is similar for near and far disparities, and they seem to be independent of stimulus contrast.

## Materials and methods

### Participants, visual acuity, and stereopsis testing

Altogether, 10 adult volunteers were involved in the study (mean age 29.18 ± 6.74 years). One participant was excluded from the analysis of Experiment 1 due to incomplete data collection. Participants were informed about the aims and course of the experiments and signed consent forms to participate. Inclusion criteria were a best corrected visual acuity of 1.0 decimal (0 logMAR) for both eyes and intact stereovision. These were tested with conventional visual acuity charts as well as Randot and TNO stereotests. All procedures were approved by the Regional and Local Research Ethics Committee of the Clinical Center at the University of Pécs (Ref.No.: 5638) and were carried out in accordance with the relevant institutional and national regulations and legislation and the World Medical Association Helsinki Declaration as revised in October 2008.

### Stimuli

The stimuli were presented on a 3D LED monitor (LG Cinema D2343P, refresh rate: 60 Hz) with polarized goggles provided by the manufacturer. Experiments were controlled by custom-made data acquisition programs in Matlab (v.R2018b, Mathworks, Natick, MA, USA) and Psychtoolbox 3.0 [39].

A rectangular presentation window of 16.4°x16.4° visual angle was filled with DRDS, a contiguous array of bright and dark square shaped “dots” each subtending 1.75’x1.75’ and refreshed at a rate of 60 Hz. Screen areas outside this window were black. The space averaged mean luminance of the stimulus area was set at 22 cd/m^2^. DRDSs were presented at two levels of Michelson-contrast (0.2 or 0.8) defined as C = (L_bright_-L_dark_)/(L_bright_+L_dark_), where L_bright_ and, L_dark_ are the luminance values of the bright and dark pixels of the DRDS stimulus, respectively.

To isolate the stereoscopic mechanism, monocular cues potentially resulting from crosstalk were minimized from the DRDSs. To this end, the luminance characteristics of the monitor were measured by an ILT1700 photometer (International Light Technologies, MA, USA) equipped with a SED033 detector, Y2 filter, and R input optic at a resolution of one step of digital value. The digital monitor values for the dark and bright pixels of the DRDS were calculated by a numerical algorithm to achieve the desired contrast and luminance levels while minimizing monocular cues [40]. The deviation from the desired values was found, with luminance and contrast errors of 0.77% and 0.36% at 80% contrast, and 0.55% and 1.16% at 20% stimulus contrast, respectively. Finally, we checked visually through the polarizing goggles that the disparity-defined stripes in the stimuli were only visible binocularly.

Reference, mask, and test stimuli were horizontal DRDS stripes of a certain disparity that subtended 1.974°×16.4° and appeared in the middle of the presentation window. Dots outside the test stimulus were always at zero disparity.

**Fig 2:**
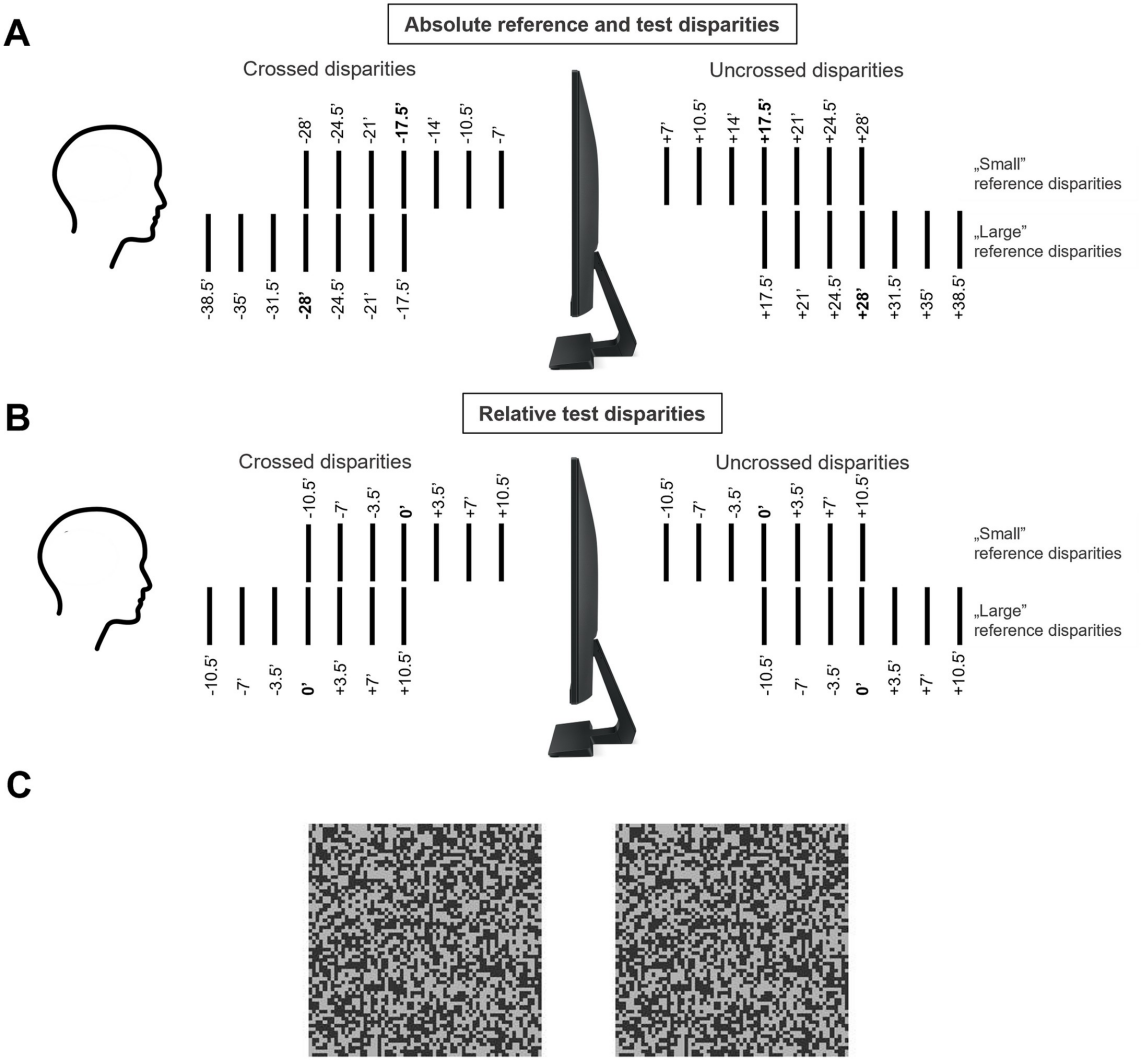
**A. Illustration of reference (indicated in bold) and corresponding test disparities in relation to the monitor and the observer expressed as absolute disparities.** The vertical lines represent the perceived order of the various disparities relative to the monitor plane. For crossed disparities (-), greater values represent stimuli closer to the observer, whereas higher uncrossed disparities (+) seem further away from the monitor plane. The top row indicates the small test disparity range (reference value: 17.5’), and the bottom row represents the higher disparity range (reference value: 28’) employed in this paradigm. **B. Illustration of reference (indicated in bold) and corresponding test disparities in relation to the monitor and the observer expressed as relative disparities.** Zero (0’) disparity represents the actual reference stimulus, and test disparities are expressed as the difference between the reference and the test disparities. **C. Stereo pair for free fusion for illustrating one of the stereograms used as reference or test stimulus in the study.** When viewed by crossed (convergent) fusion, a horizontal bar is seen in front of the image plane. Throughout the experiments all stimuli were presented on a 3D monitor and viewed through polarized goggles.

The reference stimuli had one of four disparities (reference disparities −28’, −17.5’, +17.5’ and +28’), where negative means near (crossed) and positive means far (uncrossed) disparity. The values −28’ and +28’ will also be referred to as ‘large’ disparities as compared to −17.5’ and +17.5’ called ‘small’ disparities (Fig 2A).

The disparities of test stimuli were chosen from a list of seven equally spaced values centered around the reference disparity of the current trial. Specifically, the set of test disparities relative to the reference disparity was (−10.5’, −7’, −3.5’, 0’, +3.5’, +7’, +10.5’). The reader should note that the term ‘relative test disparity’ is different from what is usually meant by ‘relative disparity’ in the literature, the latter referring to the disparity difference between two simultaneously perceived objects [17]. Absolute disparity values of the test stimuli were thus 7’, 10.5’, 14’, 17.5’, 21’, 24.5’, 28’ (for reference disparity 17.5’) or 17.5’, 21’, 24.5’, 28’, 31.5’, 35’, 38.5’ (for reference disparity 28’). The same principle applied to near disparities, only with negative sign (Fig 2). These stimulus disparities were defined in pilot experiments so that test stimuli closer to the reference were hardly distinguishable, whereas ones further away were easily discriminated by the observers (Fig 2).

Mask stimuli had absolute disparities of 0’, 7’, 12.25’ and 17.5’ (for reference disparity 17.5’) or 0’, 17.5’, 22.75’ and 28’ (for reference disparity 28’) with either positive (far) or negative (near) sign. When we refer to *relative* mask disparities, we mean |mask disparity| − |reference disparity|. Expressed this way, relative mask disparities were therefore one of the sets (0’, −5.25’, −10.5’), i.e. always closer to zero disparity than the reference stimulus. Control trials were also included, which had an interstimulus interval of 2 s without mask (‘no mask’ conditions).

### Procedure

The experiments were performed in a darkened room, from a 100 cm viewing distance. The head of the participants was positioned in a chinrest, they wore polarized goggles and were asked to keep their gaze on the central fixation mark throughout the experiment.

**Fig 3:**
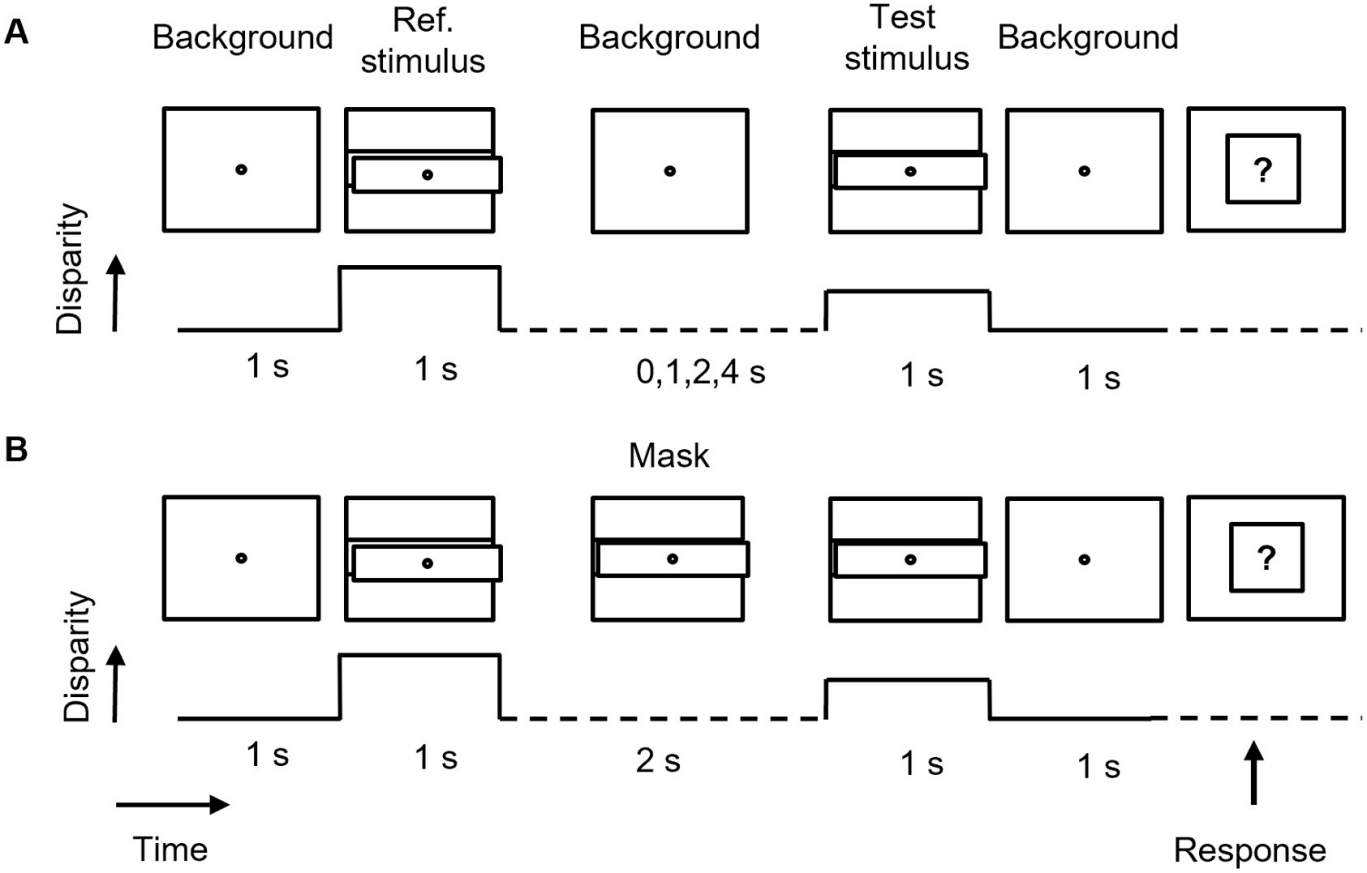
Schematic representation of the delayed disparity discrimination task. **A. No-mask experiment** (Experiment 1), **B. Masking experiment** (Experiment 2). Temporal sequence of the stimuli is indicated by the horizontal arrow. At the beginning of each trial, a reference stimulus with a predefined disparity appeared for 1 s. After a given Interstimulus Interval (ISI = 0, 1, 2 or 4 s), a test stimulus was shown, and observers were requested to indicate whether the test appeared closer or farther to the observer than the reference stimulus. Mask conditions were presented in separate blocks, whereby a 667 ms long mask stimulus of variable disparity appeared in the middle of the 2 s long ISI.

A delayed discrimination paradigm was employed together with the method of constant stimuli and the two-alternative forced choice procedure (2AFC) to measure performance. At the beginning of each trial, one of the two reference stimuli appeared for 1 s. After an interstimulus interval (ISI) of 0, 1, 2, or 4 s, the test stimulus appeared at one of the previously defined disparities for 1 s (Fig 3). In the masking conditions, only the 2 s long ISI was employed, and a mask stimulus appeared in the middle of this period for 667 ms.

The observers were asked to try to memorize the depth of the reference stimulus. At the end of each trial, when the fixation point turned into a question mark, the observer had to indicate with a button press whether the plane of the test stimulus was nearer or farther from the observer than the reference depth. Before data collection, a practice session was allowed to demonstrate the experimental procedure.

In Experiment 1, we employed the 4 references (i.e., 2 crossed and 2 uncrossed) at two contrast levels, each of which was paired with its 7 corresponding test disparity values. Every stimulus pair (reference and test) was repeated 10 times for each of the 4 ISIs. Within each block, there were only either crossed or uncrossed disparities at one contrast level, but the values that belonged to the 2 ranges and the ISI levels were mixed randomly. Each observer performed altogether 4×7×4×2×10 = 2240 (‘reference disparity’ × ‘test disparity’ × ‘ISI’ x ‘contrast’ × ‘trial repetition’) trials for no mask conditions. The trials were divided into 20, approximately 10 min long blocks, each one mixed from the small and large reference disparities, all ISIs but the same disparity sign and contrast level. Participants performed 2 to 4 of these blocks daily to minimize fatigue and learning effects.

The same parameter settings were used in the masking experiment (Experiment 2), as in the no-mask experiment. The ISI was kept constant at 2 s. Therefore, each observer performed altogether 4×7×4×2×10 = 2240 (‘reference disparity’ × ‘test disparity’ × ‘mask disparity’ × ‘contrast’ × ‘trial repetition’) trials for mask conditions. Each block was mixed from small and large reference disparities, two of the four mask conditions (the pairs were either no mask and zero relative mask disparity, or relative mask disparities −5.25’ and −10.5’, but stimuli had the same disparity sign and contrast within a block.

### Data analysis

Response accuracy was defined as the proportion of correct (“nearer” or “farther”) responses among the 10 repetitions of each combination of stimulus parameters (see above) for each participant. Response accuracy was not calculated for zero relative test disparity, because no correct response could be given when the test corresponded to the reference.

**Fig 4:**
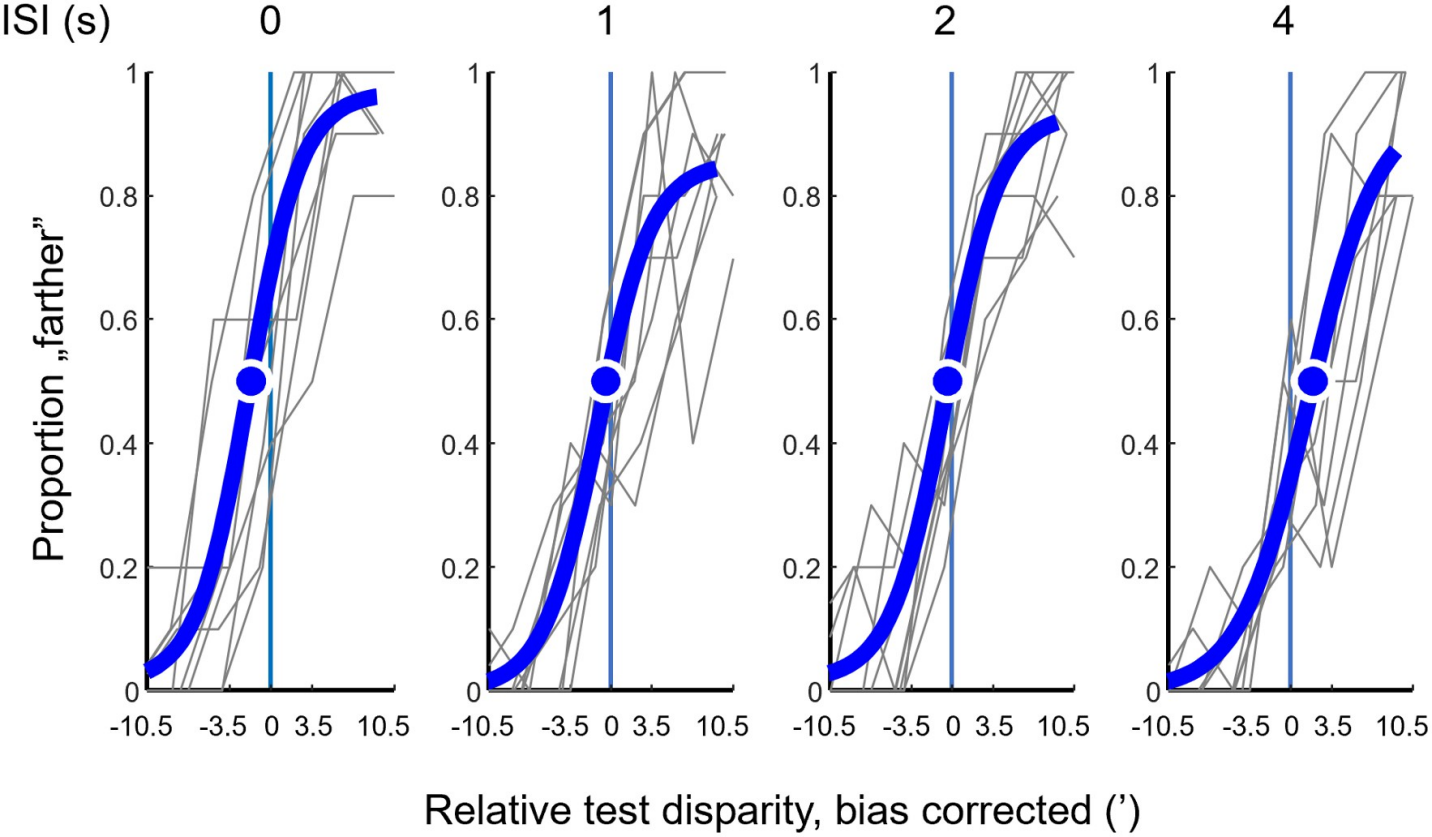
Example proportion “farther” curves (responses) as a function of relative test disparity. Data of the 9 participants (grey lines) for +17.5’ reference disparity and 0.8 contrast with the fitted logistic functions (blue). The blue dot indicates where the fitted function crosses 0.5 on the ordinate; its abscissa is the point of subjective equality (PSE). The difference of the PSE from the reference disparity (vertical blue lines) suggests a shift of remembered disparity in short-term memory. Interstimulus intervals (ISI) are shown at the top of each panel.

The proportion of “farther” responses was also expressed as a number between 0 and 1 for the 10 repetitions of each combination of stimulus parameters for each participant. Unlike response accuracy, this number could be calculated when the test corresponded to the reference. The graphs plotting the proportion of "farther” responses against relative test disparity are called the proportion farther (PF) curves (Fig 4).

The point of subjective equality (PSE) was defined as the estimated disparity (in arc min) where participants responded with equal probability that the test disparity was nearer or farther than the reference disparity. The PSE was calculated for each combination of stimulus parameters. To estimate the PSE, we first corrected the proportion farther curves for individual biases as described in the Results chapter, then fitted a logistic psychometric function to the pooled proportion “farther” responses of the 9 participants (Fig 4). For curve fitting, we used the maximum likelihood criterion as implemented in the Nelder-Mead Simplex Search algorithm in the Palamedes Toolbox for Matlab (“PAL_PFML_Fit”) [41]. All curve parameters were allowed to be optimized during the fit. The average R^2^-values of best fit logistic functions were 0.78 ± 0.096.

The following form of the logistic function was used:

$$Y\left(x,\alpha ,\beta ,\gamma ,\lambda \right)=\gamma +\left(1-\gamma -\lambda \right)\frac{1}{1+e^{-\beta (x-\alpha )}}$$ (1)

where *α* is the location of the inflexion point, *β* is the slope gradient parameter (discrimination power), *γ* and *λ* are the absolute deviations from 0 and 1 of the asymptotic values of “proportion farther” responses, respectively [41]. The PSE was finally calculated as the abscissa value (relative test disparity) where the fitted curve crossed the ordinate value of 0.5.

Standard deviations of the fitted parameters were determined by bootstrapping. Using a custom-made bootstrap function (“PAL_PFML_BootstrapNonParametric”) in Palamedes Toolbox, 100 hypothetical data sets were generated based on the measured data, each of them fitted with a logistic function and the standard deviations of the fitted parameters resulting from successful fits were calculated.

Performance was quantified based on the accuracy of responses (proportion of correct responses), and the PSE. Change of performance was expressed as a function of ISI, reference disparity, and contrast level. The results were considered significant when p<0.05. Summary data are reported as mean ± standard deviation (SD) unless otherwise stated.

## Results

### Experiment 1: Delayed discrimination

#### Accuracy of responses

**Fig 5:**
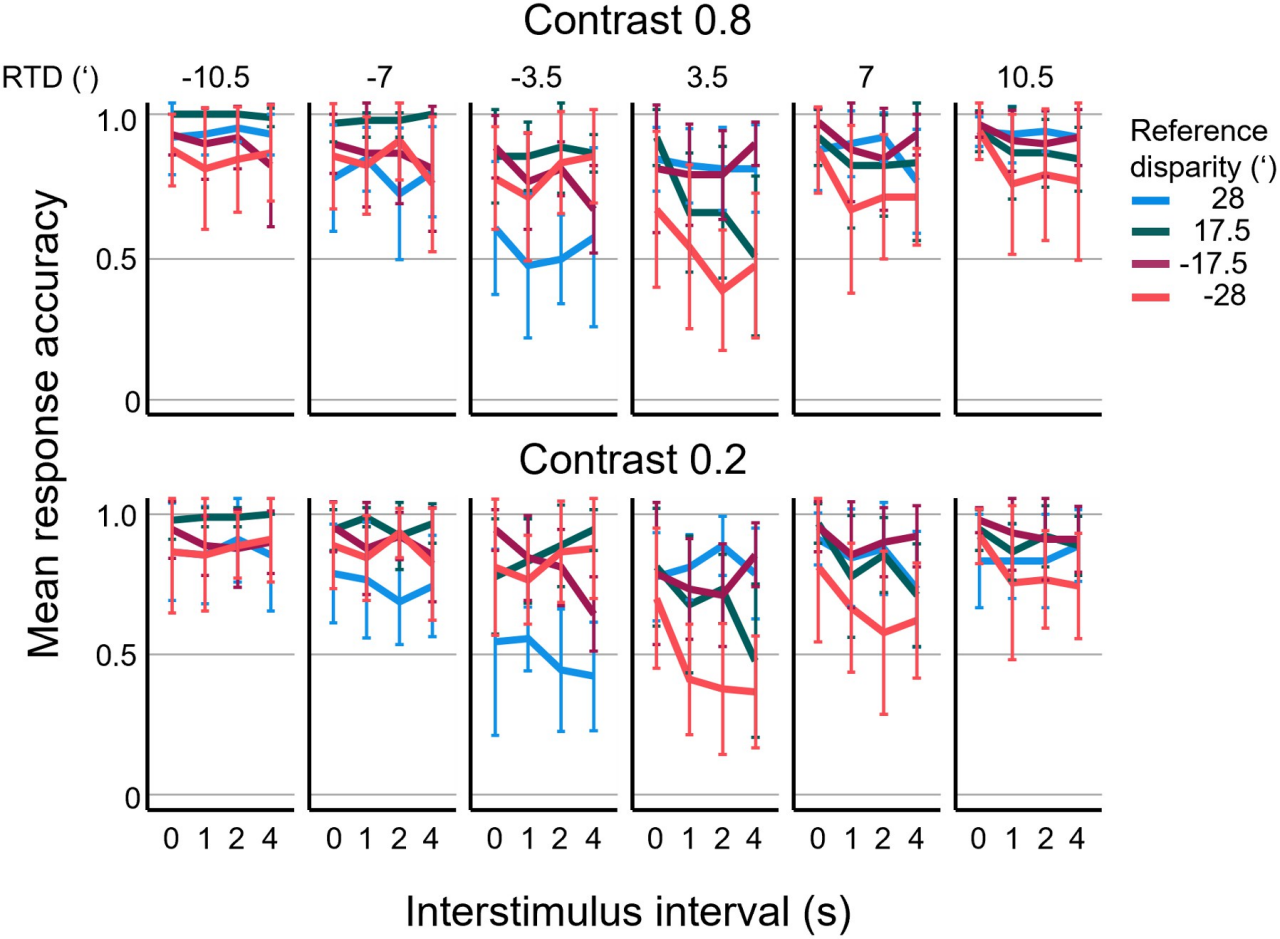
Mean accuracy (proportion correct) of responses as a function of interstimulus interval (ISI). Data for four different reference disparities (shown in different colors), at various relative test disparities (RTD, columns) and two different stimulus contrasts (rows). Test stimulus disparities are expressed relative to the reference disparity with negative values meaning nearer, and positive values meaning farther perceived depths than the reference depth, respectively. Each data point shows the mean ± SD of 9 participants.

Fig 5 shows response accuracy i.e., the proportion of correct (either “nearer” or “farther”) judgements as a function of the time participants had to remember the depth of the reference stimulus (interstimulus interval, ISI). In this representation of the data, separate plots show data points for the different relative test disparities. (For the definition of ‘relative test disparity’, see Fig 2 and Materials and methods.) The reader should note that the response accuracy at zero relative test disparity cannot be determined, because neither of the response options could be deemed correct when the test corresponded to the reference.

First, the effects of ISI, reference disparity, relative test disparity and contrast were evaluated using 4-way ANOVA. Each of the four factors had significant influence (p<0.05) with relative test disparity showing the strongest main effect (F = 72.91, η^2^_p_ = 0.18) and contrast the weakest (F = 8.020, η^2^_p_ = 0.005). The effect of relative test disparity is obvious due to the general tendency that higher disparity differences between reference and test were easier to discriminate. Nevertheless, accuracy hardly ever reached 100% confirming subjective reports of the participants about the difficulty of the task.

Accuracy dropped to a minimum when test disparity was close to the reference, indicating that in these cases, it was more difficult to discriminate reference and test disparities. For larger reference disparities (±28’), accuracy even dropped below 50%, suggesting that in certain cases, participants chose the wrong answer above chance level. The point of minimum accuracy depended on reference disparity. For example, at +28’ (uncrossed) reference disparity, the minimum accuracy was found at −3.5’ relative test disparity, whereas for −28’ (crossed) and +17.5’ (uncrossed) reference disparities, the minimum was at +3.5’ relative test disparity.

The effect of the ISI duration on accuracy indicated a decay of disparity memory with time. The first data point of each line represents ISI = 0 s, essentially a successive discrimination task without delay. Here, the accuracy depended on the disparity to be remembered, and it was generally better for smaller reference disparities (0.92 ± 0.13 and 0.93 ± 0.13 for +17.5’ and −17.5’, respectively) and worse for large reference disparities (0.81 ± 0.21 and 0.84 ± 0.20 for +28’ and −28’, respectively). Two-way ANOVA of the data for ISI = 0 s using reference disparity and test disparity as factors, followed by Tukey’s HSD (honestly significant difference) post-hoc test (*α* = 0.05) confirmed that mean accuracies were not significantly different between +17.5’ and −17.5’, or between +28’ and −28’.

When memory delay was introduced (ISI > 0 s), accuracy tended to decrease in general. This was confirmed by linear regression fitted to the response accuracies with ISI as regressor (slope = −0.015 s^−1^ ± 0.003 standard error of the mean, p < 10^−5^). When we split the data by reference disparity (different line colors in Fig 5), the linear trend was still significant with negative slopes (p<0.05) for all but +28’ reference disparity (blue lines in Fig 5).

#### Effect of the interstimulus interval on the point of subjective equality

**Fig 6:**
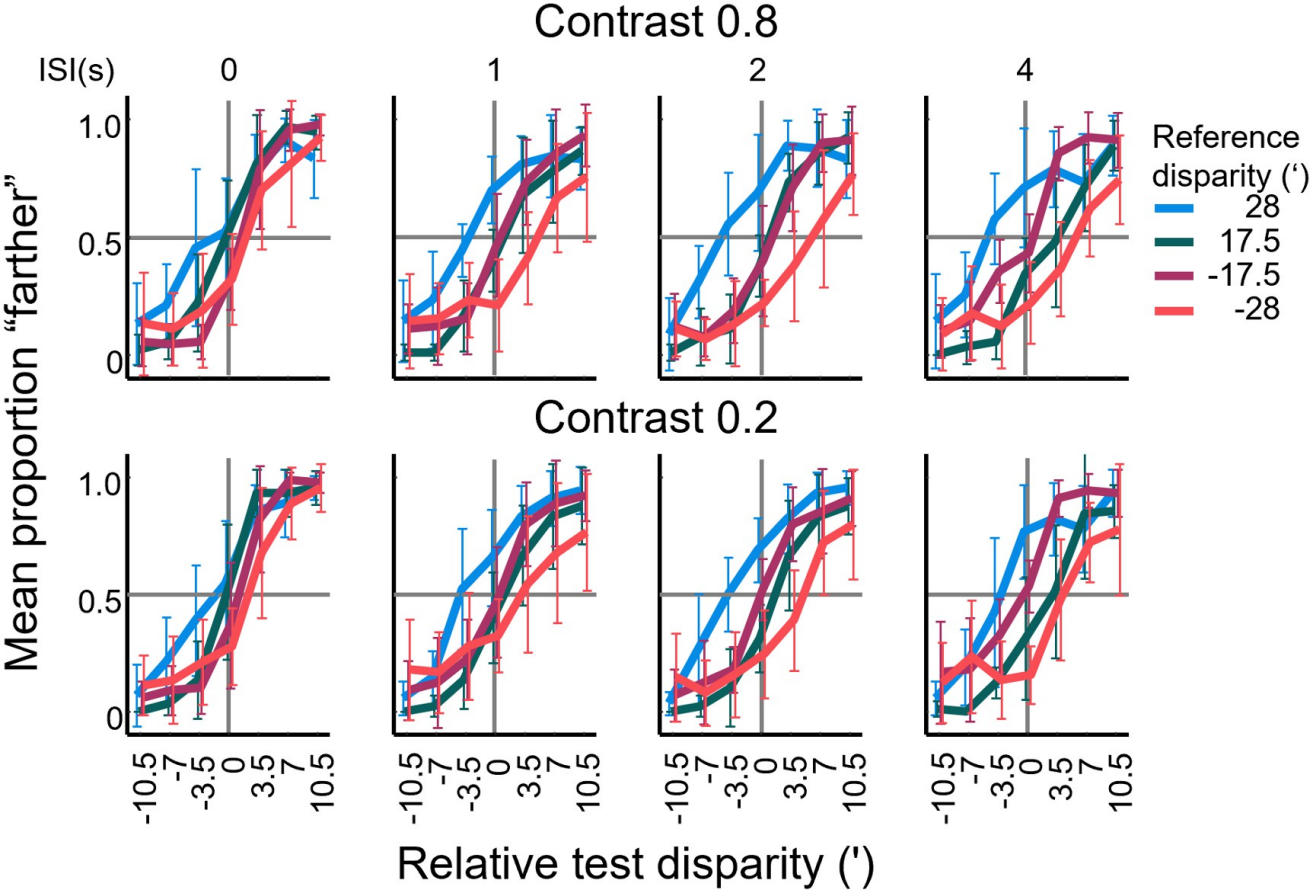
Proportion of “farther” judgements as a function of relative test disparity (PF curves) for Experiment 1. Data are shown for four different reference disparities (in different line colors), four different interstimulus intervals (ISI, increasing from left to right) and two different stimulus contrasts (different rows). Each data point shows the mean ±SD of 9 participants. Test disparity is shown relative to the reference disparity. Horizontal bold grey lines indicate 50% “farther” responses, vertical lines represent reference disparity.

Due to the nature of the forced choice procedure, participants responded either “nearer” or “farther” with a certain probability. This probability followed a largely sigmoidal course when the proportion of “farther” responses was plotted as a function of relative test disparity. We call this representation of the data the PF (proportion farther) curves hereafter (Fig 6).

In the first step of data analysis, we plotted the averaged responses of the participants. Fig 6 illustrates how the horizontal position of the sigmoid-shaped PF curves changed with ISI and reference disparity. When test disparities appeared immediately after the reference stimulus (ISI = 0), the data of the four reference disparities were running closely together. As ISI was increased however, one could observe a systematic shift in the position of the PF curves. Specifically, the curves for reference disparities of -17.5’ and +28’ moved leftwards gradually whereas those for +17.5’ and -28’ shifted rightwards.

Although it is not immediately obvious from Fig 6, we also must consider the possibility that participants had a bias in responding near over far or vice versa to the question “was the test stimulus nearer or farther than the reference stimulus” even in cases when they were in fact the same. Such response biases would add a shift to the PSE that is individual to each participant, which in turn, adds variance to the estimates of the PSE in the different experimental conditions. Assuming that the biases were constant for each individual, we pooled the proportion farther data of all conditions for each participant and fitted them with a single logistic function. The alpha parameter served as an estimate of the participant’s response bias in arc min of test disparity (0.53 ± 0.51’ across all participants). For subsequent analyses, we shifted the proportion farther curves of each participant along the horizontal axis by subtracting the value of the bias from the test disparities. This way, bias-corrected proportion farther curves of the participants could be pooled to represent the relative changes in PSE as a function of the experimental variables: reference disparity, ISI and contrast.

**Fig 7:**
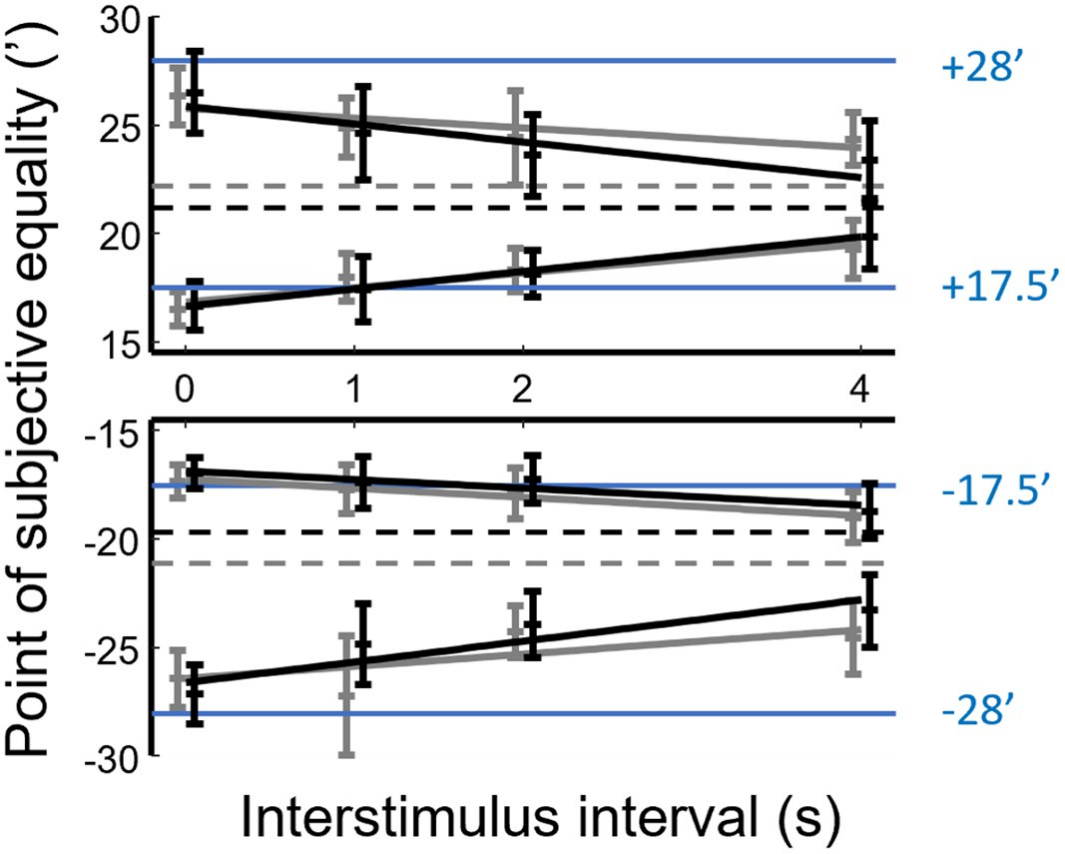
Change in PSE as a function of ISI. Data points show the mean and SD of bootstrapped data sets. Contrast levels 0.2 and 0.8 are indicated by light grey and black data points, respectively. The horizontal blue lines represent the disparity of the reference stimulus, which is also indicated by blue numbers to the right of the respective data series. Linear regression on the data points is shown by solid lines. Dashed horizontal lines indicate the PSE values at the calculated crossing of the linear trends for pairs of high and low reference disparity (from top to bottom, 22.21’, 21.21’ -19.67 and -21.07’). Separate lines are shown for each contrast level.

In the next step, we fitted the pooled data with a single logistic function (Materials and methods, Eq 1) for all participants for each combination of reference disparity, ISI, and contrast. Thus, a single PSE was obtained for each of the 32 PF datasets in Fig 6. The mean and SD of R^2^ values of the fitted curves was 0.61 ± 0.17. We defined the PSE as the test disparity where participants responded “nearer” or “farther” with equal probability. This is the point of equality between the reference disparity stored in participants’ short-term memory and the test disparity seen after the ISI. Thus, the PSE can be regarded as an estimate of the internal representation of the disparity to be remembered (Fig 1). The obtained PSE values are plotted in Fig 7, so that the ordinate shows the actual disparity values as a function of ISI. Standard deviations of the PSEs were determined by bootstrapping (see Materials and methods). Based on these plots, four observations seem worth pointing out. The first observation concerns the condition when the test stimulus appeared with zero delay. Even in this case, the PSEs had a significant bias from the reference and towards zero disparity (one-sample t-tests of the PSE shifts resulted in p<0.01 for each reference disparity and contrast combination, significance determined by Benjamini-Hochberg correction). The potential source for this initial shift will be considered later in the Discussion.

Secondly, the memory for larger disparities (±28) was more strongly affected in the task, which can be observed in Fig 7 as larger mean deviations of data points from the corresponding reference disparities (blue lines). Accordingly, when taking all non-zero ISIs and contrast levels together, the average shifts in the PSE were −3.7±1.8’ and 3.6 ± 2.0’ for the larger positive and negative disparities, respectively, whereas they were 1.0 ± 1.5’ and −0.4 ± 1.3’ for smaller (±17.5) positive and negative disparities, respectively. Due to the convention that near vs. far disparities are expressed as negative vs. positive values, the PSE shifts for the near and far versions of the same reference disparity appear to have opposite directions (see the signs of the average PSE shifts above), while they had the same direction in an absolute sense. When the PSE shifts were compared so that both near and far disparities were expressed by positive numbers, one-way ANOVA followed by the post-hoc Tukey’s HSD test revealed that the mean PSE shifts for the two small disparity conditions were significantly different from those for the large disparity conditions (p<10^−4^) but the two large disparity conditions were not different from each other (p = 0.978).

The third observation is consistent with the apparent shifts of the PF curves seen in Fig 6. As the ISI increases, we see the PSEs for larger (+28 or −28’) and smaller (+17.5 or −17.5’) reference disparities moving towards each other, and away from the original reference disparity value. Linear regression confirmed significant trends for all combinations of reference disparity and contrast (p<0.05, see trend lines in Fig 7). We chose to fit a linear trend for simplicity, although the underlying time course is likely more complex. For comparison, we fitted exponential functions (of the form *f*(*x*) = *ae*^*−bx*^ + *c*) to each of the eight time courses. We then compared the goodness of exponential fits to the corresponding linear fits by using Akaike’s Information Criterion (AIC), which compensates for the number of free parameters (2 in the linear model and 3 in the exponential model). In half of the cases, AICs were greater for the exponential model suggesting the linear model should be preferred. In three cases, the asymptote parameters for the exponential functions were far outside the biologically plausible range of disparities. As a result, we kept the more parsimonious linear model.

Since the regression lines of higher and lower reference disparities (same sign and contrast) had opposite slopes, one would expect that the PSEs reach some central value with increasing ISIs, which is largely independent of the reference disparity. We estimated this central value by finding the ordinate value of the crossing of the regression lines that belong to the higher and lower reference disparities (Fig 7). The upshot is that with time, the memory trace of disparity appears to converge towards a central value of about 20–22’ (dashed lines in Fig 7).

Finally, we analyzed whether contrast or the sign of disparity (i.e. near or far) had an effect on the slopes of the fitted linear trends (Fig 7). A steeper slope means stronger shifting of the disparity memory trace with interstimulus time, whereas a flatter slope is a sign of more stable disparity memory. We used analysis of covariance (ANCOVA) where either contrast or the sign of disparity were considered as fixed factors and ISI was modeled as a covariate. In these models, the two-way interactions between ISI and either of the factors indicate whether the rate of PSE shift changed with contrast or disparity sign.

When testing the effect of contrast, the same ANCOVA was performed separately for each reference disparity. As it is discernible from Fig 7, higher contrast resulted in steeper slopes for the larger (±28’) reference disparities (p<0.05 for the ISI × contrast interaction). However, no significant contrast effect was revealed for the smaller reference disparities (p>0.05 for the ISI × contrast interaction).

For testing near vs. far disparity differences, four separate ANCOVAs were performed for each combination of disparity magnitude (large or small) and contrast. Both near and far disparities were expressed by positive numbers. Here again, large and small disparities behaved differently. For large disparities, near and far disparities behaved symmetrically, i.e. the slopes of regression lines were statistically not different from each other, except for their opposite signs (p>0.05 for the ISI × disparity sign interaction in the case of both contrast levels). Small disparities, however, behaved asymmetrically, showing statistically steeper slopes for far than for near disparities (p<10^−4^ for the ISI × disparity sign interaction in the case of both contrast levels).

### Experiment 2: Delayed discrimination with masking

**Fig 8:**
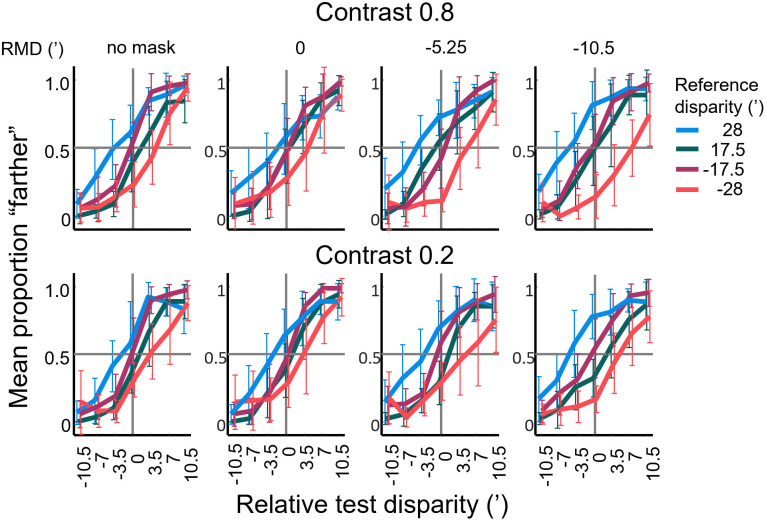
Proportion of “farther” judgements as a function of relative test disparity (PF curves) for Experiment 2. Data are shown for four different reference disparities (in different line colors), four relative mask disparities (RMD, different columns), two stimulus contrasts (different rows). Relative mask disparity equals |mask disparity| − |reference disparity|, therefore negative values mean masks closer to zero disparity than the reference stimulus. Each data point shows the mean ±SD of 9 participants. ISI was 2 s throughout. Horizontal grey lines indicate 50% “farther” responses, vertical lines represent reference disparity.

**Fig 9:**
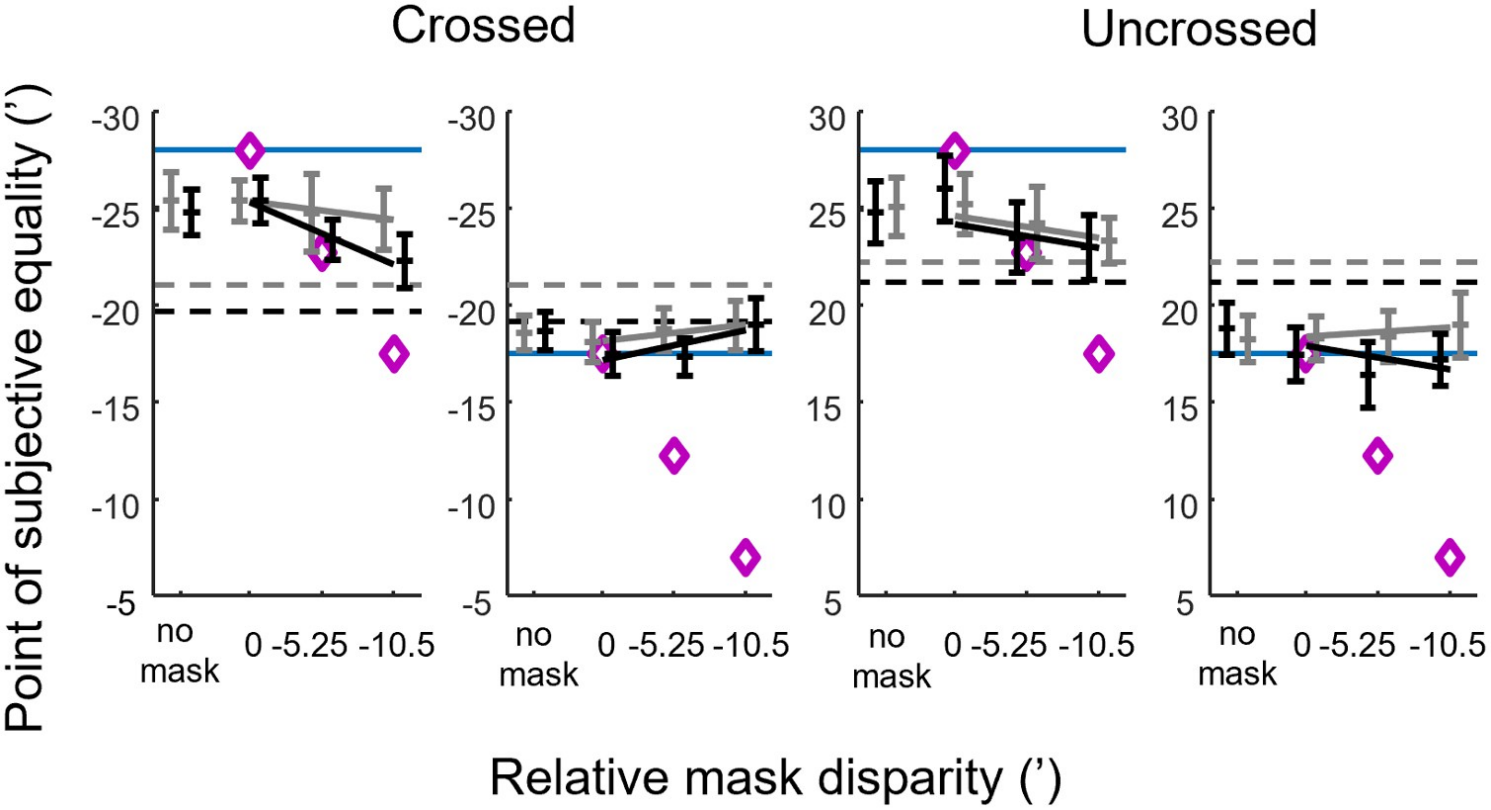
The point of subjective equality as a function of relative mask disparity. Reference disparity is indicated by the horizontal blue lines. The two panels on the left show data for uncrossed disparities, whereas those on the right show data for crossed disparities. Contrast levels 0.2 and 0.8 are indicated by light grey and black colors respectively, of the data points. Each data point shows the mean and SD of bootstrapped data sets (see Methods for details), and trend lines are linear fits.

Experiment 2 was a delayed discrimination experiment like Experiment 1, except that we presented a 667 ms long DRDS masking stimulus at half-time of the fixed 2 s interstimulus interval. We varied the disparity of the masking stimulus so that it was either equal to the reference disparity (0’ relative mask disparity) or 5.25’ or 10.5’ closer to zero disparity. In the following, we express the disparity of the mask relative to the reference disparity (i.e. 0’, −5.25’ and −10.5’ relative mask disparity, respectively). It is also worth noting that the greater the absolute value of relative mask disparity, the more dissimilar it was from the reference stimulus that had to be kept in memory. In Figs 8 and 9, the masks are shown in the order of increasing dissimilarity. Control trials were also included, which had an interstimulus interval of 2 s without mask (‘no mask’ conditions in Figs 8 and 9).

Response accuracy was again analyzed using 4-way ANOVA first, with relative mask disparity, reference disparity, relative test disparity and contrast as factors. Significant main effects were found for all factors except contrast. Relative test disparity showed the strongest effect (F = 71.65, p<0.001, η^2^_p_ = 0.22) followed by reference disparity (F = 36.65, p<0.001, η^2^_p_ = 0.08) and relative mask disparity (F = 5.61, p<0.01, η^2^_p_ = 0.009). In the following, we analyzed the effects of these variables on the points of subjective equality.

#### Effect of masking on the point of subjective equality

In Fig 8, proportion farther curves (“PF” curves) are shown for the masking experiment. As previously, the horizontal position of the sigmoid-shaped PF curves depended on reference disparity as well as on the disparity of the mask. When the mask corresponded to the reference (relative mask disparity, RMD = 0’), the data of the four reference disparities were running closely together. As the mask disparity became more dissimilar to the reference (RMD increasing in absolute value), one could observe a systematic shift in the position of the PF curves. Here, the curves for reference disparity +28’ gradually moved leftwards whereas for −28’, they gradually moved rightwards. The positions of the curves for smaller reference disparities seemed hardly affected.

As in Experiment 1, the response bias of each participant was estimated as the alpha parameter of the logistic function (Eq 1) fitted to the participant’s pooled proportion farther responses, and the bias was corrected by shifting their proportion farther curves along the horizontal axis by that amount (0.61±0.78’ across all participants). The bias-corrected proportion farther curves of the participants were pooled to represent the relative changes in PSE as a function of the experimental variables: reference disparity, relative mask disparity and contrast. We then fitted the pooled data of all participants with a single logistic function (Eq 1) for each of the 32 PF datasets in Fig 8. The mean and SD of R^2^ values of the fitted curves was 0.69 ± 0.19. Again, the test disparity where participants responded “nearer” or “farther” with equal probability was taken as estimate of the PSE.

Fig 9 summarizes the changes of the PSE in a similar way as it was done for Experiment 1 (Fig 7), but here, the abscissa shows the relative mask disparity in the order of increasing dissimilarity from the reference. The four reference disparities are organized in separate panels in Fig 9 to increase clarity. As we will see below, the effect of increasingly dissimilar masks was generally analogous to, but somewhat more complex than, the effect of increasing ISI. As for Experiment 1, our observations are organized in four points.

It is clear from Fig 9 that the PSE was already significantly shifted away from the corresponding reference disparity (blue lines) in the baseline “no mask” condition (one-sample t-tests of the PSE shifts resulted in p<10^−4^ for each reference disparity and contrast combination, significance determined by Benjamini-Hochberg correction). This is to be expected, since this condition is essentially a repetition of the 2 s ISI case from Experiment 1, even though in a different context. Similarly, the direction of the shifts is towards the hypothetical ‘default disparity’ (dashed lines).

Secondly, the application of a memory mask seemed to enhance this tendency, most strongly for the larger reference disparities (1^st^ and 3^rd^ panels in Fig 9). When we pooled all mask and contrast combinations, one-way ANOVA with reference disparity as the explanatory variable, showed a significant effect (p<0.05) on the PSE shift values. According to the post-hoc Tukey’s HSD test, far and near versions of the larger reference disparity were not significantly different from each other (p = 0.264) with average (unsigned) PSE shifts of -3.55 ± 1.73’ and -3.80 ± 1.93’, respectively. The smaller reference disparities (±17.5’) were however, affected significantly less (p<0.001) with PSE shifts of only -0.30 ± 1.64’ and 0.78 ± 1.32’, respectively.

The third observation was that more dissimilar memory masks increased the shifts in PSE, but this effect was convincing only for reference disparities of ±28’. Here, the PSEs appeared to move towards the disparity of the mask (purple diamonds in Fig 9) indicating a ‘pulling effect’. For small reference disparities (2^nd^ and 4^th^ panels in Fig 9) a slight but monotonous change of PSE can be observed in most data series, but a pulling effect of the mask is not seen. To model the PSE shift, a simple linear trend was assumed for simplicity. Regression was done separately for each reference disparity and contrast combination. Linear fits were reasonable for larger disparities (R^2^ 0.28 ± 0.20, p<0.05), but they were weak for small reference disparities (R^2^ 0.09 ± 0.09, p<0.05 except for +17.5’ at 80% contrast), consistent with generally less effect of the mask for the latter.

Finally, the effect of contrast and near vs. far disparities was analyzed on the steepness of the fitted linear trends by using analysis of covariance. The rationale of this analysis is to test how strongly the disparity memory may be perturbed by an increasingly dissimilar memory mask under different stimulus conditions. The analysis was essentially the same as for Experiment 1, except that we used relative mask disparity as the covariate instead of ISI.

The effect of contrast (indicated by line color in Fig 9) was tested separately for each reference disparity. Altogether, the contrast effect lacked consistent patterns. For three of the four reference disparities (-28’, -17.5’ and +17.5, indicated by the blue lines in the respective panels), a significant mask disparity × contrast interaction was seen (p<0.05), but the direction and magnitude of the effect was diverse.

A near vs. far comparison of the slopes painted a similarly complex picture. To judge these differences visually, one should compare the data either from the 1^st^ and 3^rd^ panels, or from the 2^nd^ and 4^th^ panels in Fig 9. Out of the four combinations of disparity magnitude and contrast, three showed no statistically significant differences between near and far versions of the same disparity (p>0.05 for the interaction of mask disparity and disparity sign). The exception was the ±17.5’ reference at high contrast, but as we have seen above, the linear trends were weak or non-significant for these reference disparities.

## Discussion

This study reports data on visual short-term memory for disparity, employing the traditional psychophysical method of delayed discrimination and memory masking. Using inter-stimulus intervals (ISIs) of up to 4 s, a range of disparities, two contrast levels and masking stimuli, we quantified the decay of stored information. Our study was the first to our knowledge to test VSTM for stereo depth using random dot stereograms, by which we could examine the storage of depth cues in isolation from monocularly visible spatial cues.

### How bad is disparity memory?

In summary, we found that increasing memory delay significantly decreased accuracy, which was explained by a systematic shift in the point of subjective equality with ISI. PSE values for higher and lower disparities, regardless of sign, moved towards a central value (around 20–22’, Fig 7), away from the original reference disparity. The application of mask stimuli also altered the PSE, with the magnitude of this shift typically increasing with the dissimilarity between the mask and the stimulus to be remembered (Fig 9). The direction of mask-induced shifts was mostly identical to the shifts evoked by increasing ISI. The pattern of these changes was qualitatively similar for crossed and uncrossed disparities, although the rates of change in PSE were different in some cases. Both time delay and masking exhibited a more pronounced effect on the retention of larger (crossed as well as uncrossed) disparities, i.e. memory for smaller disparities was more robust. Finally, the above effects were largely independent of or lacked systematic relationship to stimulus contrast.

Visual short-term memory for stereoscopic depth has gained relatively little attention before. Studies by Xu and Nakayama [42] and Reeves and Lei [43] found that placing memory items at different stereoscopic depth planes had no advantage for memory performance, unless remembering depth position was explicitly required. The latter result pointed to the existence of a memory buffer for depth itself (Visual Working Memory for depth, VWMd), and subsequent studies began testing properties of this memory mechanism. It has repeatedly been found that VWMd is less precise than memory for planar visual cues [44–46]. Quian and Zhang [44] measured that participants could detect a change in depth of a single visual item correctly in 78% of the cases. Comparable data from our measurements can be inferred from trials where the difference between reference and test disparities was ±10.5’ and the ISI was 1 s (Fig 5). Depending on the reference disparity, our participants reached mean accuracies between 74–99% (mean of all participants 89±16%) in these cases, similar to those of Qian and Zhang [44]. Further support for the inaccuracy of VSTM for depth came from our observation that participants’ depth judgements were significantly biased even when test items were presented immediately after the reference.

Whereas remembering absolute disparity is clearly difficult, memory performance improves significantly when the relative depth positions of multiple items have to be recalled [47].

The main difference between earlier analyses and the present study is that here, we aimed to test disparity memory in isolation from other potential cues such as shape, color, spatial location etc. To achieve this, the reference and test items had identical spatial arrangements (they were both horizontal bars) and locations, and we made sure that the only perceptible change during a trial was that of stereoscopic depth by using random dot stereograms.

### The effect of ISI

The gradual shift in PSE with storage time was in line with many of the earlier data about storage properties of visual information such as spatial frequency, color, orientation, contrast and the direction and speed of motion in VSTM [2, 5, 7, 16]. However, it is also clear from these studies, that the accuracy of storage depends on the feature to be remembered. For example, the short-term storage of orientation, spatial frequency and velocity are highly accurate whereas contrast, direction of movement and color show some sort of decay with increasing storage time [3, 5, 6, 9, 13]. Our results altogether indicate that disparity information too, gradually loses its fidelity with increasing memory duration [6, 7].

Regarding the time course of storage in VSTM, Magnussen [48] found that information processing goes through two phases, which was evident in a change in choice reaction times (although not in accuracy) with increasing interstimulus interval. In another study, Reeves and Lei [45] found that the ease of recalling numerals based on their stereoscopic depth also follows a biphasic time course during the first 2 s of retention, although their task likely invoked spatial, semantic as well as disparity memory mechanisms. The shift in PSE in our data was sufficiently approximated by a linear trend with a constant slope, although the underlying process is certainly more complex. For instance, PSE would likely reach a plateau instead of drifting indefinitely with time. A more detailed theoretical model would however depend on the postulated underlying mechanism, which should involve the buildup of a representation in visual memory including perceptual averaging (see below and [35, 48–51]) as well as memory decay.

### Effect of disparity

Numerous studies have already shown an advantage in the processing of crossed against uncrossed disparities, for example in stereoacuity [52] and simple reaction times [22, 23, 52, 53]. Qian et al. [54] added depth cues to their memory items in a test of VSTM for color and size and found that memory performance was better for objects perceived in front of the fixation plane than for those behind it. Here, we tested memory for stereoscopic depth in the absence of other task relevant cues and found no systematic advantage for near disparities.

On the other hand, disparity magnitude did have an effect: both the increase in ISI and the application of masking stimuli affected the storage of larger (±28’) disparities (both crossed and uncrossed) stronger (Figs 7 and 9). This difference may originate in the differences between processing of fine (up to around 20’) and coarse disparities [30]. Such a dichotomy is also supported by clinical observations that fine and coarse stereopsis can be selectively lost while the other function still remains intact [55]. Since the larger reference disparities of our experiments were already in the coarse disparity range, the results suggest that VSTM for such disparities suffers from stronger decay during retention. The change in the PSE may have been magnified by increased disparity discrimination thresholds at larger disparities, which can reach approximately 2’ for pedestal disparities of 30’ versus a threshold of about 1’ for 20’ pedestals [56]. Indeed, participants already showed significant PSE shifts at zero ISI (Fig 7) suggesting that the direction of even immediate changes relative to large disparities (±28’) were difficult to judge correctly whereas the shift in PSE was minor or non-significant relative to small references (±17.5’). The question remains, however, what determines the direction of such biases. One potential source for this initial shift is that perception of the test stimuli is biased by the reference stimulus immediately preceding it.

### Basis of the “default” disparity

The general observation from our data is that the memory trace of disparity is gradually replaced by what we call a “default” depth value of about 20–22’ when the memory task becomes increasingly difficult either due to increased retention time or following dissimilar masking stimuli. In the following we consider the potential roles of the disparity tuning of the visual system and perceptual averaging influencing the default depth value.

It is clear that the visual system is biologically optimized (tuned) to process and encode disparity information that aligns with naturally occurring and ecologically relevant disparities [57, 58]. Disparity tuning curves of the human stereoscopic system have been obtained by using visual evoked potentials [59–61], functional magnetic resonance imaging [62] or by measuring simple reaction times [22]. The upshot of these studies is that the peak of the neuronal response is between 10 and 16’ and responses decline towards smaller as well as greater disparities. If the memory buffer for disparity was also tuned to this range, it could result in memory representations gravitating towards the optimum sensitivity of the memory store in cases of uncertainty. Our results could be taken as evidence for such preferred memory representation, however, a default disparity between 10–16’ is unlikely to explain our results (Figs 7 and 9).

An alternative explanation could be based on psychophysical data suggesting that there is a temporal or spatial averaging of perceived and stored information in the visual system [32, 63, 64]. One potential benefit of such a mechanism is that representing the summary statistics of visual (or other perceptual) objects in memory is more economical than storing properties of individual items. Furthermore, biasing the response towards the average during recall might improve performance in situations of uncertainty [32, 34, 35, 65].

The relative difficulty of our VSTM paradigm (Fig 5) raises the possibility that perceptual averaging biased participants’ decisions. Calculated from the set of stimuli (including reference and test) presented in one experimental session of Experiment 1 (see Materials and methods), the arithmetic mean disparity was 22.75’, not far from the measured central values of 20–22’. Uncertainty was naturally increased with increasing interstimulus interval, thus the observation that PSEs converged towards those central values is compatible with the theory of perceptual averaging.

There has been a single study that attempted to quantify the bias of stereoscopic depth in stored VSTM [66]. The authors interpreted their results as a “contraction bias” meaning that the remembered disparity latently shifted towards zero disparity during retention. This finding seems to contradict our results where we saw the bias moving towards values around 20–22’. The two studies can be reconciled, however, if we consider that the stimulus set of Zhang et al. [66] contained negative (near) and positive (far) disparities symmetrically within the same session, and thus, their perceptual average was zero. Indeed, the same authors found later [67] that perceptual averaging can explain the results of Zhang [66].

The outcome of our masking experiment is more difficult to reconcile with this idea. The average disparity of the stimulus set was 21.44’ when taking into account the (task irrelevant) masking stimuli. It is, however, less clear which of the conditions could be regarded more difficult than the other, except that having no mask in the interstimulus interval certainly causes less perturbation in VSTM than any mask. Huang and Sekuler [36] have demonstrated that task-irrelevant stimuli alongside the reference elevate uncertainty and do induce perceptual averaging. Indeed, it seems that applying increasingly dissimilar masks “pulled” the PSE towards a central value in Experiment 2 that is around the average and would thus be compatible with larger uncertainty (Fig 9).

The synthesis of these two concepts provides a comprehensive framework for understanding disparity memory dynamics. It integrates the biological predispositions of the visual system with its adaptive response strategies, offering a holistic view of how disparity information is processed and retained in memory.

This study has provided insights into the mechanisms of visual short-term memory for disparity. Although human memory for absolute disparity has proven to be imprecise and volatile, its tendency towards perceptual averages supports adaptation of the visual system to stimuli in the environment.

## Supporting information

S1 Data (XLSX)

S2 Data (XLSX)
